# Supplementary material for: Large-scale characterisation of the nasal microbiome redefines Staphylococcus aureus colonisation status
Source: Nat Commun. 2025 Dec 2;16:10415. doi: 10.1038/s41467-025-66564-4 (PMC12672568; doi:10.1038/s41467-025-66564-4)
Supplement: Supplementary file 1 — Supplementary Information [file 41467_2025_66564_MOESM1_ESM.pdf]

Supplementary Materials for Aggarwal *et al*.

Supplementary Tables

Name	Sequence
V1FW_SD501	AATGATACGGCGACCACCGAGATCTACACAAGCAGCAacactctttccctacacgacgctcttccgatctNNNNAGMGTTYGATYMTGGCTCAG
V1FW_SD502	AATGATACGGCGACCACCGAGATCTACACACGCGTGAacactctttccctacacgacgctcttccgatctNNNNAGMGTTYGATYMTGGCTCAG
V1FW_SD503	AATGATACGGCGACCACCGAGATCTACACCGATCTACacactctttccctacacgacgctcttccgatctNNNNAGMGTTYGATYMTGGCTCAG
V1FW_SD504	AATGATACGGCGACCACCGAGATCTACACTGCGTCACacactctttccctacacgacgctcttccgatctNNNNAGMGTTYGATYMTGGCTCAG
V1FW_SD505	AATGATACGGCGACCACCGAGATCTACACGTCTAGTGacactctttccctacacgacgctcttccgatctNNNNAGMGTTYGATYMTGGCTCAG
V1FW_SD506	AATGATACGGCGACCACCGAGATCTACACCTAGTATGacactctttccctacacgacgctcttccgatctNNNNAGMGTTYGATYMTGGCTCAG
V1FW_SD507	AATGATACGGCGACCACCGAGATCTACACGATAGCGTacactctttccctacacgacgctcttccgatctNNNNAGMGTTYGATYMTGGCTCAG
V1FW_SD508	AATGATACGGCGACCACCGAGATCTACACTCTACACTacactctttccctacacgacgctcttccgatctNNNNAGMGTTYGATYMTGGCTCAG
V2RV_SD701	CAAGCAGAAGACGGCATACGAGATACCTAGTAgtgactggagttcagacgtgtgctcttccgatctNNNNGCTGCCTCCCGTAGGAGT
V2RV_SD702	CAAGCAGAAGACGGCATACGAGATACGTACGTgtgactggagttcagacgtgtgctcttccgatctNNNNGCTGCCTCCCGTAGGAGT
V2RV_SD703	CAAGCAGAAGACGGCATACGAGATATATCGCGgtgactggagttcagacgtgtgctcttccgatctNNNNGCTGCCTCCCGTAGGAGT
V2RV_SD704	CAAGCAGAAGACGGCATACGAGATCACGATAGgtgactggagttcagacgtgtgctcttccgatctNNNNGCTGCCTCCCGTAGGAGT
V2RV_SD705	CAAGCAGAAGACGGCATACGAGATCGTATCGCgtgactggagttcagacgtgtgctcttccgatctNNNNGCTGCCTCCCGTAGGAGT
V2RV_SD706	CAAGCAGAAGACGGCATACGAGATCTGCGACTgtgactggagttcagacgtgtgctcttccgatctNNNNGCTGCCTCCCGTAGGAGT
V2RV_SD707	CAAGCAGAAGACGGCATACGAGATGCTGTAACgtgactggagttcagacgtgtgctcttccgatctNNNNGCTGCCTCCCGTAGGAGT
V2RV_SD708	CAAGCAGAAGACGGCATACGAGATGGACGTTAgtgactggagttcagacgtgtgctcttccgatctNNNNGCTGCCTCCCGTAGGAGT
V2RV_SD709	CAAGCAGAAGACGGCATACGAGATGGTCGTAGgtgactggagttcagacgtgtgctcttccgatctNNNNGCTGCCTCCCGTAGGAGT
V2RV_SD710	CAAGCAGAAGACGGCATACGAGATTAAGTCTCgtgactggagttcagacgtgtgctcttccgatctNNNNGCTGCCTCCCGTAGGAGT
V2RV_SD711	CAAGCAGAAGACGGCATACGAGATTACACAGTgtgactggagttcagacgtgtgctcttccgatctNNNNGCTGCCTCCCGTAGGAGT
V2RV_SD712	CAAGCAGAAGACGGCATACGAGATTTGACGCAgtgactggagttcagacgtgtgctcttccgatctNNNNGCTGCCTCCCGTAGGAGT

Supplementary Table S1: Full length Illumina tagged primers used in study

**Original taxa**	**BLAST assigned taxa**	**OTU**
Solanum melongena (eggplant)	*Moraxella osloensis*	7278
Ambystoma mexicanum (axolotl)	*Acinetobacter lwoffii*	16873
gamma proteobacterium enrichment culture clone JF9-3	*Pseudomonas stutzeri*	7869
Ceuthophilus sp.	*Pseudomonas sp.*	17786
bacterium enrichment culture clone Guo10	*Acinetobacter sp.*	16545
uncultured gamma proteobacterium	*Acinetobacter johnsonii*	16234
uncultured gamma proteobacterium	*Acinetobacter sp.*	6386
Coregonus lavaretus (common whitefish)	*Cutibacterium acnes*	1449
uncultured gamma proteobacterium	*Pseudomonas sp.*	15694
uncultured alpha proteobacterium	*Agrobacterium sp.*	2447
Athetis lepigone	*Pseudomonas putida*	15973
uncultured gamma proteobacterium	*Pantoea agglomerans*	19430
bacterium enrichment culture clone Guo10	*Acinetobacter johnsonii*	16549
Triticum aestivum (bread wheat)	*Pantoea agglomerans*	1713
Cyanobacteria/Melainabacteria group bacterium S15B-MN24 CBMW_12	*bacterium*	2448
Triticum aestivum (bread wheat)	*Pseudomonas sp.*	14880
uncultured gamma proteobacterium	*Acinetobacter sp.*	22396
uncultured organism	*Streptococcus sp.*	18567
Bacteroidetes endosymbiont of Metaseiulus occidentalis	*Sphingobacterium multivorum*	18113
uncultured Xanthomonadales bacterium	*Stenotrophomonas sp.*	24764
uncultured Kocuria sp.	*Kocuria sp.*	11727
uncultured Magnetococcus sp.	*Magnetococcus sp.*	17306
uncultured organism	*Morganella morganii*	13974
uncultured Stenotrophomonas sp.	*Stenotrophomonas sp.*	14445
Solanum melongena (eggplant)	*Moraxella osloensis*	7664
uncultured Frigoribacterium sp.	*Frigoribacterium sp.*	8729
uncultured gamma proteobacterium	*Acinetobacter sp.*	22692
Serratia plymuthica RVH1	*Serratia plymuthica*	12905
uncultured organism	*Streptococcus sp.*	9583
uncultured gamma proteobacterium	*Pseudomonas sp.*	14885
uncultured organism	*Streptococcus sp.*	18518
uncultured organism	*Streptococcus sp.*	18519
uncultured proteobacterium	*Moraxella osloensis*	7312

uncultured organism	*Stenotrophomonas sp.*	24912
Kocuria palustris PEL	*Kocuria palustris*	4869
Antarctic seawater bacterium Bsw10170	*Psychrobacter sp.*	8875
bacterium enrichment culture clone Guo10	*Acinetobacter sp.*	6395
uncultured organism	*Streptococcus sp.*	18569
uncultured organism	*Streptococcus sp.*	9603
uncultured organism	*Morganella morganii*	13970
Lepisosteus oculatus (spotted gar)	*Cutibacterium sp.*	26069
halophilic and alkaliphilic bacterium HN24	*Alteribacillus sp.*	4290
uncultured Bacteroidetes bacterium	*Sphingobacterium multivorum*	8736
uncultured organism	*bacterium*	26105
Trichuris trichiura (human whipworm)	*bacterium*	9363
Lepisosteus oculatus (spotted gar)	*bacterium*	11552
gamma proteobacterium symbiont of Graphosoma rubrolineatum	*Pantoea sp.*	19435
uncultured organism	*Streptococcus sp.*	10002
uncultured gamma proteobacterium	*Pseudomonas sp.*	7868
uncultured organism	*Segatella copri*	11683
uncultured beta proteobacterium	*Massilia sp.*	15297
Sargassum henslowianum	*Pseudomonas sp.*	15771
Type-C symbiont of Plautia stali	*Pantoea dispersa*	11877
Frankliniella occidentalis (western flower thrips)	*Erwinia sp.*	20685
uncultured organism	*Haemophilus parainfluenzae*	13484
uncultured organism	*Lactobacillus gasseri*	4438
uncultured organism	*Staphylococcus sp.*	5125
uncultured organism	*Staphylococcus aureus*	14222
unidentified	*Streptococcus sp.*	6344
Ceuthophilus sp.	*Pseudomonas oleovorans*	23177
uncultured organism	*bacterium*	18471
uncultured organism	*Escherichia coli*	26623
bacterium 1A5	*Chryseobacterium oncorhynchi*	1979
uncultured organism	*Stenotrophomonas maltophilia*	17898
uncultured organism	*Streptococcus sp.*	9588
uncultured microorganism	*Helcococcus sp.*	8325
uncultured alpha proteobacterium	*Agrobacterium sp.*	26581
Triticum urartu	*bacterium*	4985
Trichuris trichiura (human whipworm)	*bacterium*	9366

unidentified marine bacterioplankton	*Salmonella enterica*	21359
human gut metagenome	*bacterium*	3703
uncultured organism	*Streptococcus sp.*	4119
uncultured gamma proteobacterium	*Acinetobacter radioresistens*	22712
uncultured organism	*Staphylococcus sp.*	5119
uncultured organism	*bacterium*	17984
uncultured organism	*bacterium*	17911
Coregonus lavaretus (common whitefish)	*Cutibacterium acnes*	20637
uncultured organism	*Neisseria sp.*	26003
uncultured organism	*Lactobacillus crispatus*	3520
Triticum aestivum (bread wheat)	*bacterium*	11971
Teleogryllus commodus	*Pseudomonas sp.*	24751
uncultured organism	*Staphylococcus aureus*	15046
uncultured organism	*Streptococcus sp.*	18501
Trichuris trichiura (human whipworm)	*bacterium*	9368
unidentified marine bacterioplankton	*bacterium*	12169
Solanum torvum	*Paracoccus sp.*	12792
halophilic and alkaliphilic bacterium HN24	*Dolosigranulum pigrum*	14546
Coregonus lavaretus (common whitefish)	*Cutibacterium acnes*	1457
Ambystoma mexicanum (axolotl)	*Acinetobacter lwoffii*	26268
Solanum melongena (eggplant)	*Moraxella osloensis*	26387
Solanum melongena (eggplant)	*Moraxella osloensis*	7286
uncultured organism	*Stenotrophomonas sp.*	25790
uncultured alpha proteobacterium	*Agrobacterium sp.*	26410
Teleogryllus commodus	*Enterobacteriaceae bacterium*	4745
halophilic and alkaliphilic bacterium HN24	*Alteribacillus sp.*	14551
uncultured organism	*Acinetobacter sp.*	15599
uncultured organism	*Stenotrophomonas sp.*	25791
uncultured eubacterium WD264	*bacterium*	6823
halophilic and alkaliphilic bacterium HN24	*Alteribacillus sp.*	21604
uncultured organism	*bacterium*	9586
uncultured organism	*bacterium*	17934
uncultured organism	*Morganella morganii*	21474
bacterium enrichment culture clone Guo10	*Acinetobacter sp.*	26388
Ceuthophilus sp.	*Pseudomonas sp.*	26487
uncultured gamma proteobacterium	*Pseudomonas sp.*	15975
uncultured organism	*Granulicatella adiacens*	6828
Solanum melongena (eggplant)	*Moraxella osloensis*	26075

Eogystia hippophaecolus	*Acinetobacter guillouiae*	25143
Ambystoma mexicanum (axolotl)	*Acinetobacter lwoffii*	26305
uncultured organism	*Morganella morganii*	13077
Ceuthophilus sp.	*Pseudomonas sp.*	26560
Ambystoma mexicanum (axolotl)	*Acinetobacter lwoffii*	26024
Coregonus lavaretus (common whitefish)	*Cutibacterium acnes*	26118
uncultured cyanobacterium	*bacterium*	26032
uncultured gamma proteobacterium	*Acinetobacter johnsonii*	22462
Ambystoma mexicanum (axolotl)	*Acinetobacter lwoffii*	16338
bacterium enrichment culture clone Guo10	*Acinetobacter sp.*	6394
uncultured organism	*Staphylococcus sp.*	10024
unidentified marine bacterioplankton	*Lactobacillales group*	3609
BTEX-degrading bacterium enrichment culture	*Stenotrophomonas maltophilia*	66
uncultured organism	*bacterium*	17943
halophilic and alkaliphilic bacterium HN24	*Alteribacillus sp.*	21605
Trichuris trichiura (human whipworm)	*bacterium*	18332
uncultured compost bacterium	*Corynebacterium sp.*	934
uncultured gamma proteobacterium	*Acinetobacter johnsonii*	22448
Ceuthophilus sp.	*Pseudomonas sp.*	26431
Ambystoma mexicanum (axolotl)	*Acinetobacter lwoffii*	26026
uncultured organism	*bacterium*	10382
uncultured organism	*bacterium*	17988
Solanum melongena (eggplant)	*Moraxella osloensis*	26025
Ambystoma mexicanum (axolotl)	*Acinetobacter lwoffii*	26584
uncultured gamma proteobacterium	*Acinetobacter johnsonii*	26005
gamma proteobacterium enrichment culture clone JF9-3	*Pseudomonas stutzeri*	26035
uncultured organism	*Staphylococcus sp.*	14223
uncultured gamma proteobacterium	*Acinetobacter sp.*	26554
Solanum melongena (eggplant)	*Moraxella osloensis*	25996
uncultured gamma proteobacterium	*Acinetobacter sp.*	16057
human gut metagenome	*Faecalibacterium prausnitzii*	8225
Triticum aestivum (bread wheat)	*Pantoea agglomerans*	26244
Ambystoma mexicanum (axolotl)	*Acinetobacter lwoffii*	26100
Mycobacterium tuberculosis	*Rothia mucilaginosa*	4908
Mycobacterium tuberculosis	*Rothia mucilaginosa*	4903

Supplementary Table S2: Mis-assigned taxa by ARB. A few operational taxonomic units were evaluated with BLAST where the species was disconcordant with the taxa genus and/or not a prokaryote, as assigned by ARB, or where an

uncultured bacterium was seen across the mock microbial community suggesting it was either an expected mock microbial community member or a significant contaminant.

Species	Low DNA Qubit R1	Low DNA Qubit R2	Extraction Location Associated	PCR Location Associated	Known Contaminant Correlation R1	Known Contaminant Correlation R2	Decontam (neg control)	Biological Plausibility	Contaminant
S. bongori	No	No	NA	NA	NA	NA	Possible	No	Yes
S. aureus	No	No	No	No	Yes	No	Possible	Yes	No
D. pigrum	No	No	No	No	No	No	Possible	Yes	No
Corynebacterium sp.	No	No	No	No	No	No	Possible	Yes	No
C pseudodiphtheriticum	No	No	No	No	No	No	Unlikely	Yes	No
C. jeikeium	No	No	No	No	No	No	Unlikely	Yes	No
C. accolens	No	No	No	No	No	No	Unlikely	Yes	No
C. koseri	No	No	No	No	Yes	No	Possible	Yes	No
K. michiganensis	No	No	No	No	No	No	Possible	Yes	No
S. epidermidis	No	Yes	No	No	No	No	Unlikely	Yes	No
K. pneumoniae	No	No	No	No	Yes	No	Unlikely	Yes	No
B. cereus	No	No	No	No	Yes	No	Possible	No	Yes
K. oxytoca	No	No	No	No	Yes*	No	Possible	Yes	No
Acinetobacter sp.	No	No	No	No	Yes	Yes	Possible	No	Yes
M. catarrhalis	No	No	No	No	No	No	Possible	Yes	No
K. aerogenes	No	No	No	No	No	No	Unlikely	Yes	No
A. ursingii	No	No	No	No	Yes	Yes*	Possible	No	Yes
Acinetobacter sp.	No	No	No	No	Yes	Yes	Possible	No	Yes
E. coli	No	No	No	No	Yes	Yes	Possible	Yes	No
C. freundii	No	No	No	Yes	No	Yes	Possible	Yes	No
Pseudomonas sp.	No	No	No	Yes	Yes	Yes*	Possible	No	Yes
M. osloensis	No	No	No	No	No	No	Unlikely	Yes	No
Psychrobacter sp.	No	No	No	No	Yes*	No	Possible	No	Yes
A. lwoffii	No	No	No	No	Yes*	Yes	Possible	No	Yes
M. nonliquefaciens	No	No	No	No	Yes	No	Possible	Yes	No
Rhodococcus sp.	No	No	No	No	Yes	No	Unlikely	No	Yes

Enhydrobacter sp.	No	No	Yes	No	No	Yes	Unlikely	No	Yes
Alkanindiges sp.	No	No	No	No	No	No	Unlikely	No	Yes
P. stutzeri	No	No	No	No	Yes	Yes	Possible	No	Yes
S. marcescens	No	No	No	No	No	No	Possible	Yes	No
M. lincolnii	No	No	No	No	Yes	Yes	Unlikely	Yes	No
Chryseobacterium sp.	No	No	No	Yes	No	Yes	Possible	No	Yes
Ralstonia sp.	No	Yes	No	Yes	Yes	Yes*	Unlikely	No	Yes
Pseudomonas sp.	No	No	No	Yes	Yes	Yes*	Possible	No	Yes
Enterobacter sp.	No	No	No	Yes	Yes*	Yes	Possible	No	Yes
A. johnsonii	No	No	No	No	Yes*	Yes	Unlikely	No	Yes
Stenotrophomonas sp.	No	No	No	No	Yes*	Yes*	Possible	No	Yes
P. mirabilis	No	No	No	No	Yes	No	Unlikely	No	Yes
Magnetococcus sp.	No	No	No	No	Yes	No	Unlikely	No	Yes
E. faecalis	No	No	No	No	Yes	Yes	Unlikely	No	Yes
P. coleopterorum	No	No	No	No	Yes	No	Possible	No	Yes
A. parvus	No	No	No	No	No	Yes	Unlikely	No	Yes
A. johnsonii	No	No	No	No	Yes*	Yes	Unlikely	No	Yes
M. lacunata	No	No	No	No	No	Yes	Unlikely	Yes	No
P. proteolytica	No	No	No	No	Yes	Yes	Unlikely	No	Yes
P. gergoviae	No	No	No	No	Yes	Yes	Possible	No	Yes
R. mucilaginosa	No	No	No	No	No	No	Unlikely	Yes	No
S. haemolyticus	No	No	No	No	Yes	No	Unlikely	Yes	No
S. maltophilia	No	No	Yes	No	Yes	Yes*	Possible	No	Yes
E. cloacae	No	No	No	No	Yes	Yes	Unlikely	No	Yes
S. sciuri	No	No	No	No	No	No	Unlikely	Yes	No
P. agglomerans	No	No	No	No	Yes	Yes	Unlikely	No	Yes
P. aeruginosa	No	No	Yes	Yes	Yes*	Yes	Possible	No	Yes
Streptococcus sp.	No	No	No	No	Yes	Yes	Unlikely	Yes	No
P. putida	No	No	No	No	Yes*	Yes	Possible	No	Yes
Klebsiella sp.	No	No	No	No	Yes*	Yes	Unlikely	Yes	No

M. morganii	No	No	No	No	No	No	Unlikely	Yes	No
C. acnes	No	Yes	No	No	Yes	Yes	Unlikely	Yes	No
P. oryzihabitans	No	No	NA	NA	No	Yes	Unlikely	No	Yes
M. morganii	No	No	No	No	No	Yes	Unlikely	Yes	No
P. frederiksbergensis	No	No	No	No	Yes	Yes	Unlikely	No	Yes
R. terrigena	No	No	No	No	No	Yes	Possible	No	Yes
S. dysgalactiae	No	No	No	No	No	No	Unlikely	Yes	No
Stenotrophomonas sp.	No	No	No	No	Yes*	Yes*	Possible	No	Yes
Moraxella sp.	No	No	No	No	No	No	Unlikely	Yes	No

Supplementary Table S3: Determination of possible contaminants from low biomass anterior nares' samples.

Species	Comparison	Log-fold change	Standard error	adjusted p-value
Corynebacterium accolens	Persistent ~ Non-carrier	-0.95	0.30	0.03
Corynebacterium jeikeium	Persistent ~ Non-carrier	-1.40	0.29	<0.001
Corynebacterium sp.	Persistent ~ Non-carrier	-1.66	0.28	<0.001
Dolosigranulum pigrum	Persistent ~ Non-carrier	-1.63	0.29	<0.001
Moraxella catarrhalis	Persistent ~ Non-carrier	-0.64	0.21	0.04
Staphylococcus aureus	Persistent ~ Non-carrier	5.50	0.24	<0.001
Staphylococcus aureus	Intermittent ~ Non-carrier	1.45	0.29	<0.001
Staphylococcus epidermidis	Persistent ~ Non-carrier	-0.75	0.23	0.02

Supplementary Table S4: Differential abundance of species by nasal colonisation status using ANCOM-BC2 displaying significant associations. Log fold (natural log) changes as compared to *Staphylococcus aureus* non-carriers. Log-fold change between the compared groups shown with associated standard error and adjusted p-values. P-values adjusted with holm method.

**Intercept**	**Odds Ratio**	**Lower C.I**	**Upper C.I.**
CST II	0.59	0.37	0.95
CST III	0.86	0.56	1.32
CST IV	0.64	0.40	1.02
CST V	0.22	0.11	0.43
CST VI	0.50	0.30	0.84
CST VII	2.00	1.41	2.84
**Sex**	**Odds Ratio**	**Lower C.I**	**Upper C.I.**
CST II	0.63	0.38	1.04
CST III	0.70	0.45	1.09
CST IV	0.69	0.42	1.14
CST V	1.73	0.88	3.42
CST VI	0.53*	0.30	0.93
CST VII	0.67*	0.47	0.96
**Current Smoker**	**Odds Ratio**	**Lower C.I**	**Upper C.I.**
CST II	0.59	0.16	2.17
CST III	0.86	0.31	2.42
CST IV	0.60	0.16	2.24
CST V	1.00	0.21	4.76
CST VI	0.79	0.21	2.94
CST VII	0.73	0.31	1.70
**Pets**	**Odds Ratio**	**Lower C.I**	**Upper C.I.**
CST II	1.13	0.68	1.86
CST III	0.87	0.55	1.36
CST IV	0.94	0.56	1.56
CST V	0.68	0.35	1.35
CST VI	1.08	0.62	1.87
CST VII	0.93	0.65	1.33
**Healthcare contact**	**Odds Ratio**	**Lower C.I**	**Upper C.I.**
CST II	0.87	0.39	1.94
CST III	1.07	0.54	2.10
CST IV	0.71	0.30	1.67
CST V	0.86	0.28	2.67

CST VI	0.77	0.31	1.90
CST VII	0.90	0.52	1.58
**Chronic Skin Condition**	**Odds Ratio**	**Lower C.I**	**Upper C.I.**
CST II	1.00	0.45	2.20
CST III	1.09	0.55	2.15
CST IV	0.79	0.34	1.86
CST V	1.30	0.49	3.44
CST VI	0.89	0.36	2.19
CST VII	1.21	0.70	2.08
**Asthma**	**Odds Ratio**	**Lower C.I**	**Upper C.I.**
CST II	1.05	0.41	2.69
CST III	0.88	0.38	2.07
CST IV	1.28	0.52	3.15
CST V	0.00	0.00	2.32e+201
CST VI	0.76	0.24	2.38
CST VII	0.62	0.30	1.27
**Allergy**	**Odds Ratio**	**Lower C.I**	**Upper C.I.**
CST II	0.73	0.42	1.24
CST III	0.77	0.48	1.23
CST IV	0.69	0.40	1.19
CST V	0.73	0.36	1.46
CST VI	0.79	0.44	1.43
CST VII	0.90	0.62	1.30

Supplementary Table S5: Multinomial logistic regression model of major lifestyle and clinical risk factors for *S. aureus* Community State Types. Odds ratios are presented with 95% Confidence Intervals (C.I.). We found men had a reduced relative risk for association with CST VI (OR = 0.53, 95% CI = 0.30-0.93, p=0.03) and CST VII (OR=0.67, 95% CI= 0.47-0.96, p=0.03) compared with CST1. * p<0.05, ** p<0.01, *** p<0.001.

Taxon	Group	Eigenvector value (2 d.p.)
Serratia marcescens	0	5.09e-17
Morganella morganii subsp.	0	1.96e-17
Klebsiella aerogenes	0	0
Dolosigranulum pigrum	1	1
Moraxella catarrhalis	1	0.71
Corynebacterium jeikeium	1	0.70
Corynebacterium pseudodiphtheriticum	1	0.66
Corynebacterium accolens	1	0.54
Moraxella nonliquefaciens	1	0.34
Moraxella lincolnii	1	0.32
Klebsiella oxytoca	1	0.23
Staphylococcus aureus	2	0.79
Corynebacterium sp.	2	0.69
Cutibacterium acnes	2	0.64
Staphylococcus epidermidis	2	0.62
Rothia mucilaginosa	2	0.42
Moraxella osloensis	2	0.28
Moraxella sp.	2	0.06
Citrobacter freundii	3	0.07
Klebsiella michiganensis	3	0.01
Escherichia coli	3	0.01
Moraxella lacunata	3	0.01
Klebsiella pneumoniae	4	9.88e-17
Citrobacter koseri	4	5.38e-17

Supplementary Table S6: Eigenvector centrality values from network analysis. Species-level networks were inferred with NetCoMi (v1.2) using SparCC correlations (zeroes replaced by a pseudocount; centred log-ratio transform; 1000 bootstraps), then analysed for centrality and community structure via fast-greedy clustering

Supplementary Figures

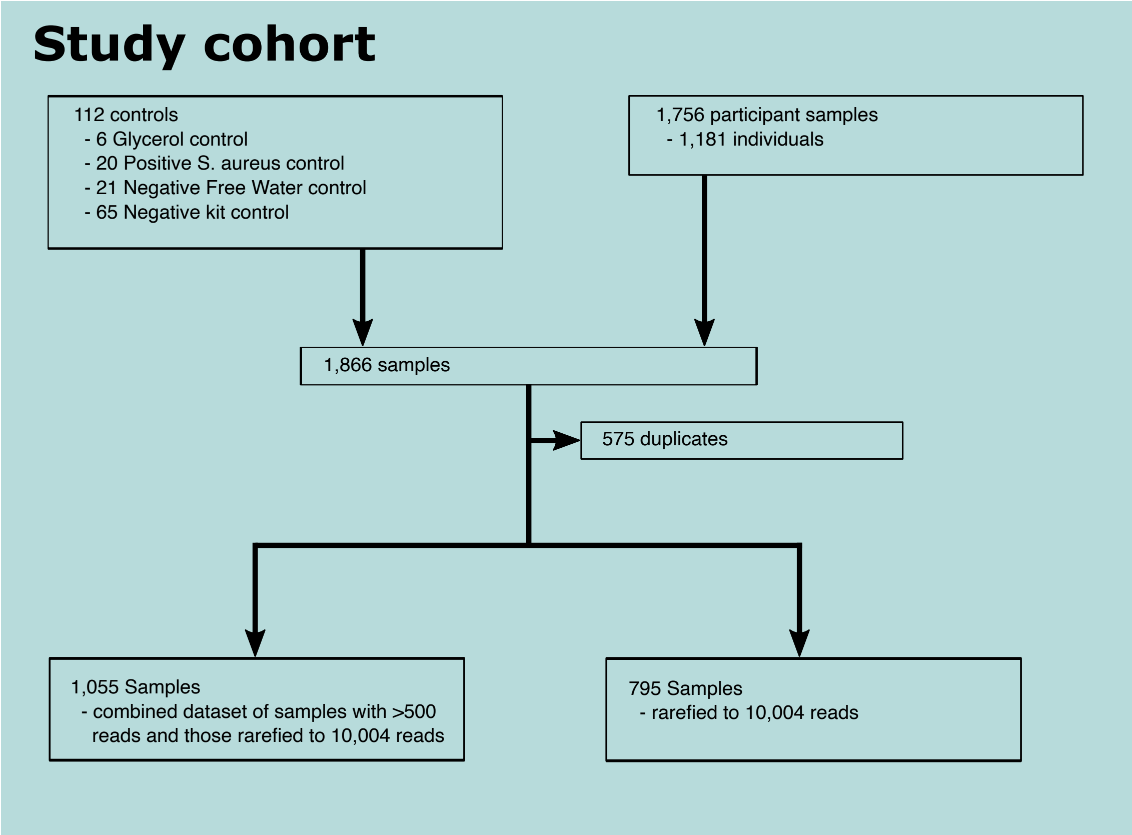

Supplementary Fig. S1: Study cohort and available 16S rRNA sequences.

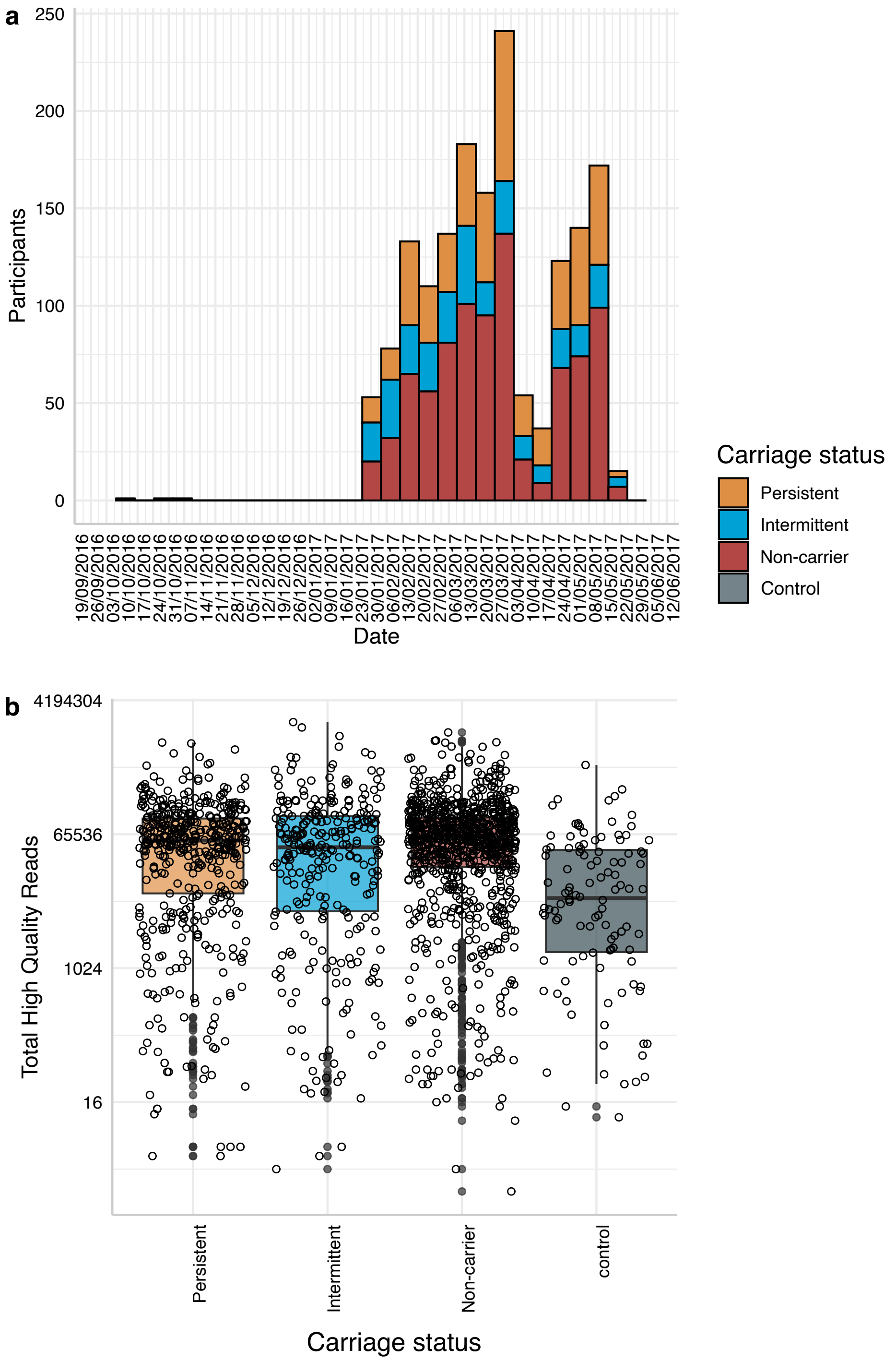

Supplementary Fig. S2: Nasal samples processed for 16S rRNA gene sequencing from the CARRIAGE study with high-quality reads after mothur processing. (a) all samples processed over the study period (b) box plot to show variation in the total read count (i.e. 16S rDNA gene copies) by carriage or control status. For (a) and (b), n=1866 samples.

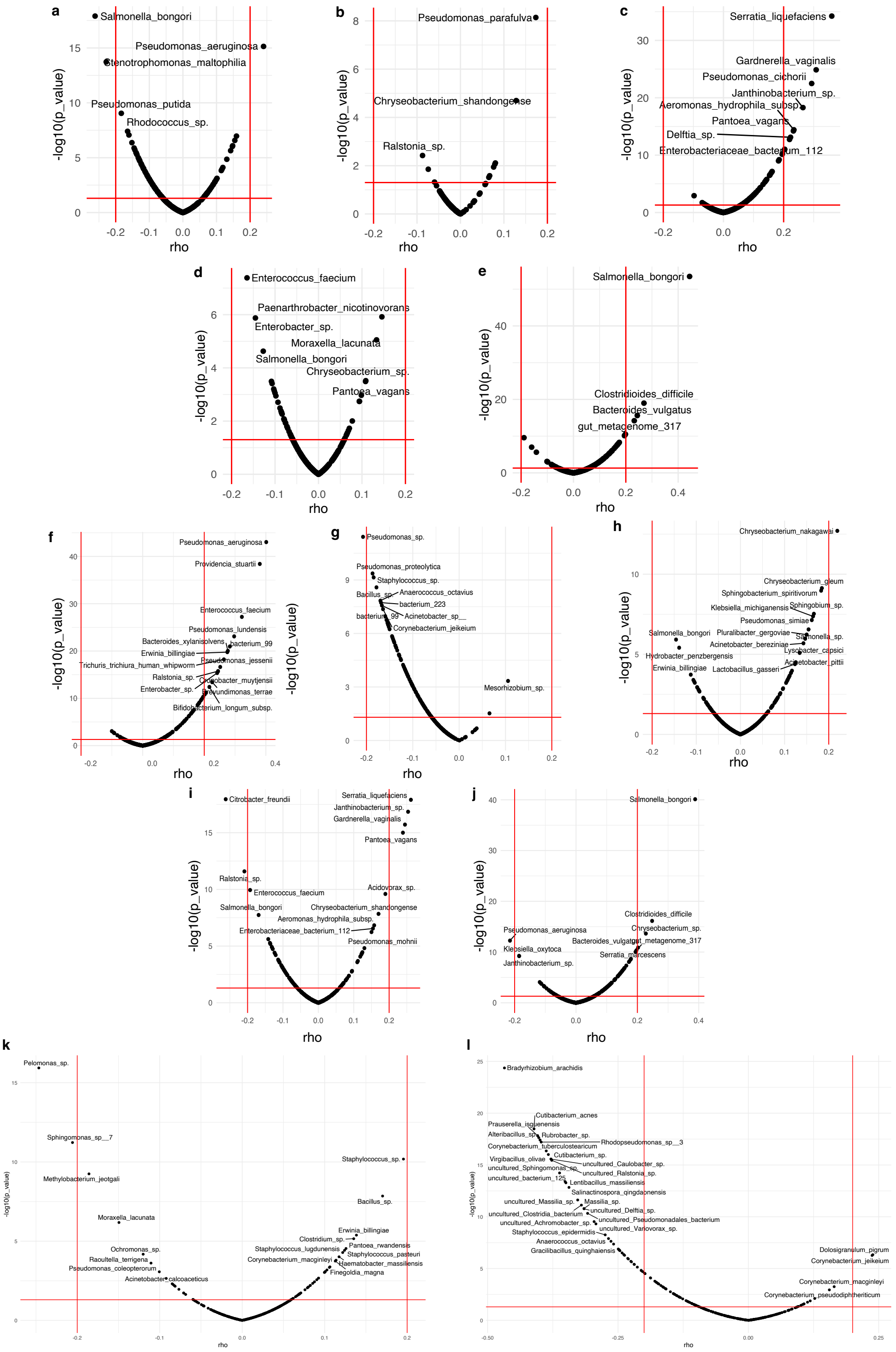

Supplementary Fig. S3: Identification of contaminants through batch effects and association with sample DNA concentration. Y-axis represents log 10 of p-value of spearman's correlation coefficient calculation, and horizontal 'red-line' intercepts y-axis at p=0.05. Vertical red-line intercepts spearman rho values at -0.2 and 0.2. **(a-e)** correlation of taxa relative abundance at a species level with each extraction location where samples from the first batch were processed. **(f-j)** correlation of taxa relative abundance at a species level with each PCR amplification location where samples

from the first batch were processed, n=1,099 samples. **(k-l)** correlation of taxa relative abundance at a species level with DNA concentration measurement (qubit) after PCR amplification from the first batch of samples processed **(k)**, n=1,099 samples, and the second batch of samples processed **(l)**, n=767 samples.

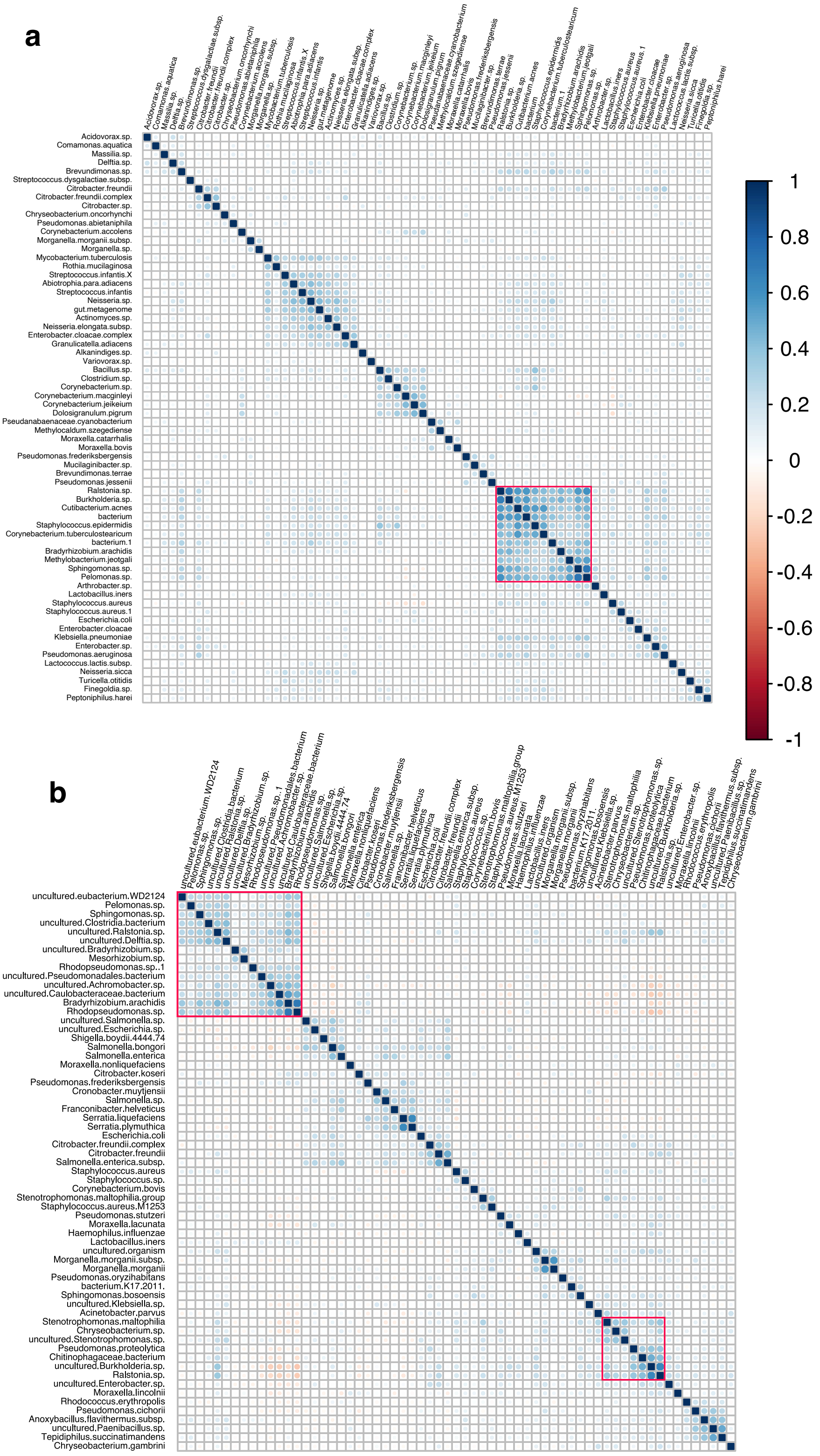

Supplementary Fig. S4: Heatmap demonstrating correlation of contaminants with one another. All species were correlated with one another and ordered with

hierarchical clustering of the spearman's correlation coefficient rho values. Only significant correlation values are displayed ($p<0.05$). The correlation heatmap was then subset to allow for visualisation of examples that displayed clusters involving known contaminants that are highlighted with the red boxes. **(a)** subset plot from species-to-species correlation plot of the first batch of samples processed (n=1099 samples). **(b)** subset plot from species-to-species correlation plot of the second batch of samples processed (n=767 samples).

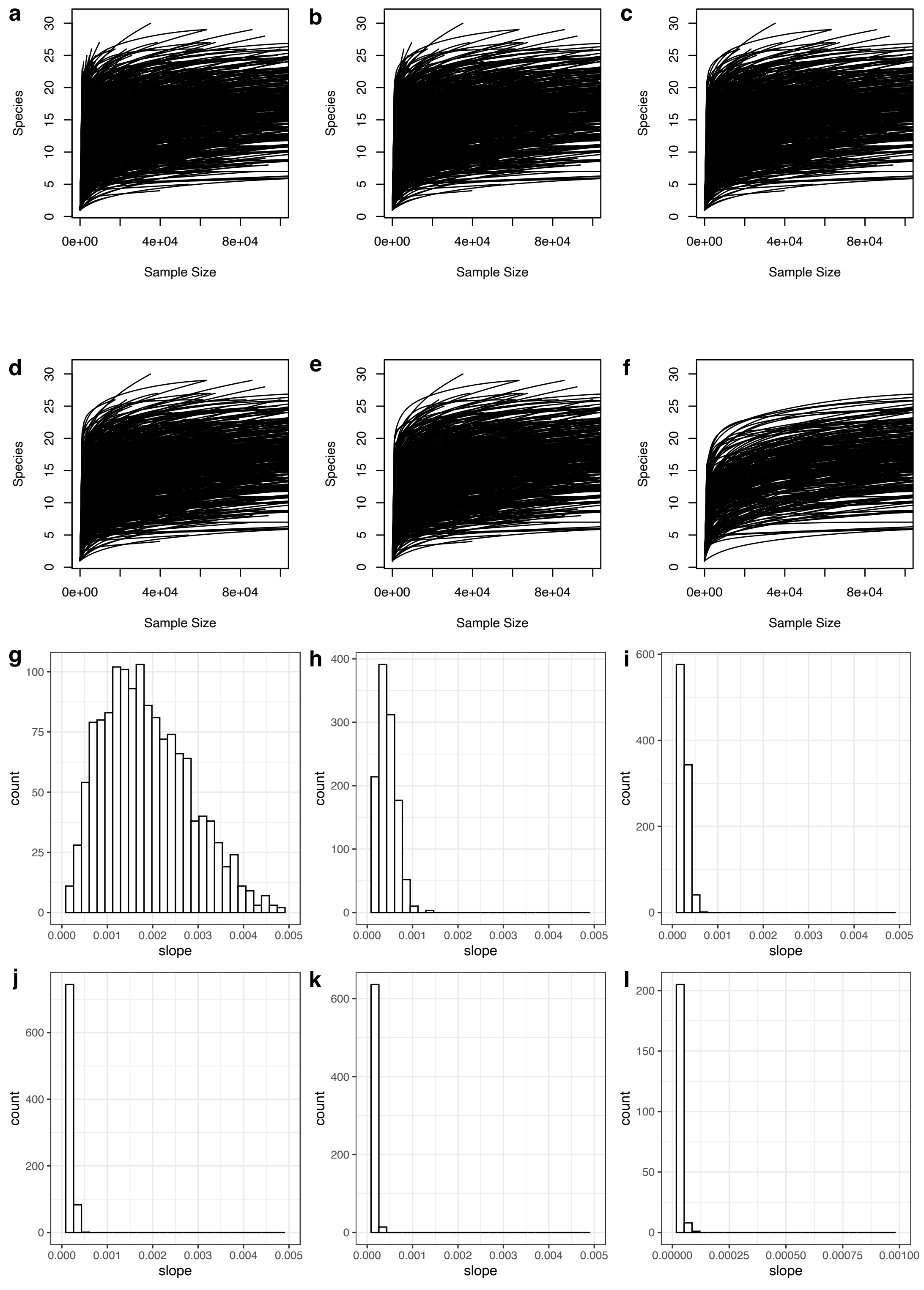

Supplementary Fig. S5: Setting a rarefication threshold. (a-f) Rarefication curves generated for samples (n=1,756 samples) after the removal of samples with less than a) 1,000 b) 5,000 c) 10,000 d) 15,000 e) 20,000 and f) 100,000 high-quality reads. **(g-l)** Histogram representing the slope of the rarefaction curves generated in figures **a to f** at the respective high-quality read count threshold below which samples were removed g) 1,000 h) 5,000 i) 10,000 j) 15,000 k) 20,000 and fl 100,000 high-quality reads.

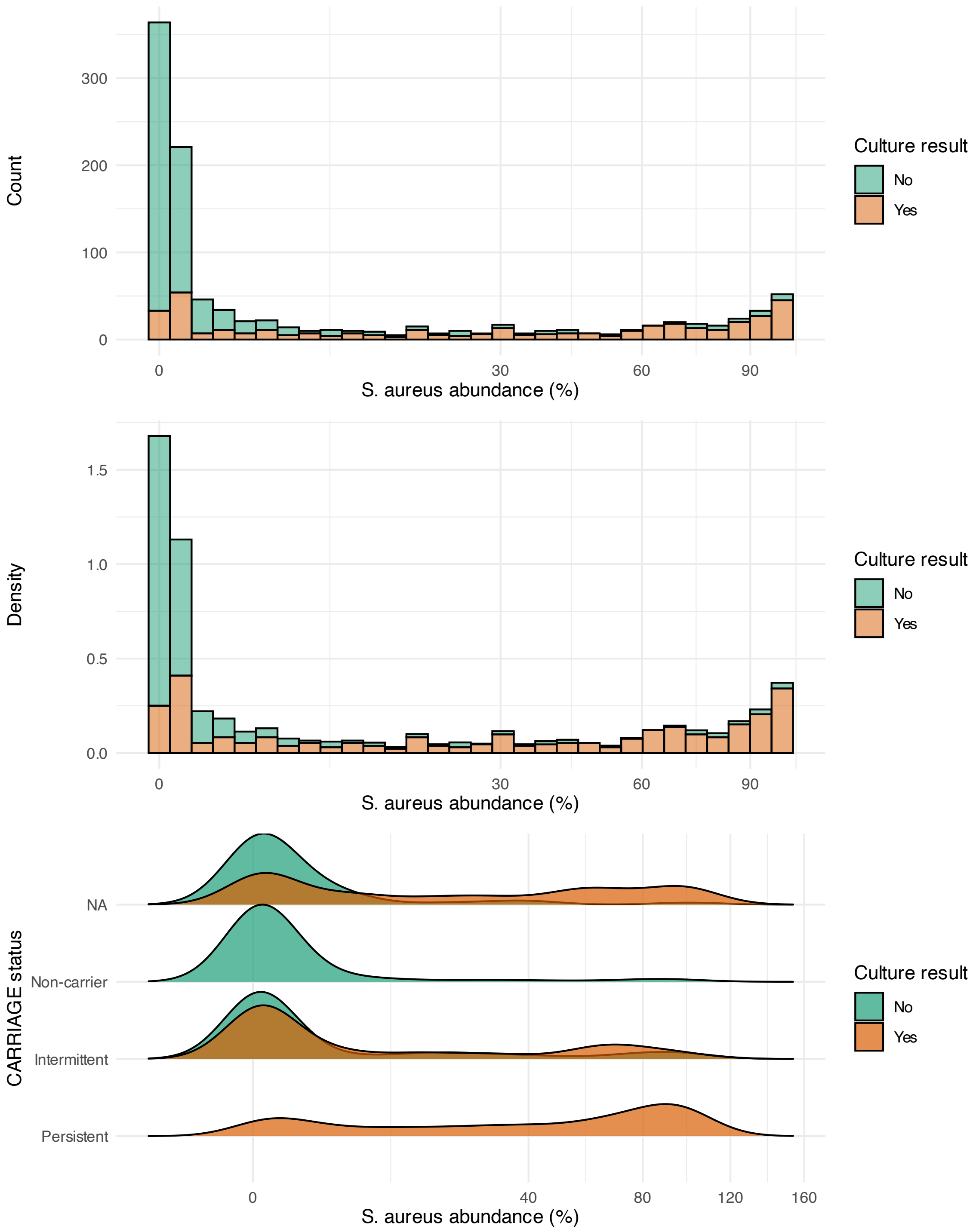

Supplementary Fig. S6: Relationship of *Staphylococcus aureus* culture and *S. aureus* abundance (a) Histogram distribution (counts) of *S. aureus* abundance (%) by culture result. Negative culture, n=672 samples, Positive culture, n=382 samples. (b) Density distribution of *S. aureus* abundance (%) by culture result. Negative culture, n=672 samples, Positive culture, n=382 samples. (c) Ridgeline densities of *S. aureus* abundance (%) across non-, intermittent-, and persistent- colonisation states and culture outcomes: persistent, n=275 samples; intermittent, n=169 samples; non-carrier, n=532 samples.

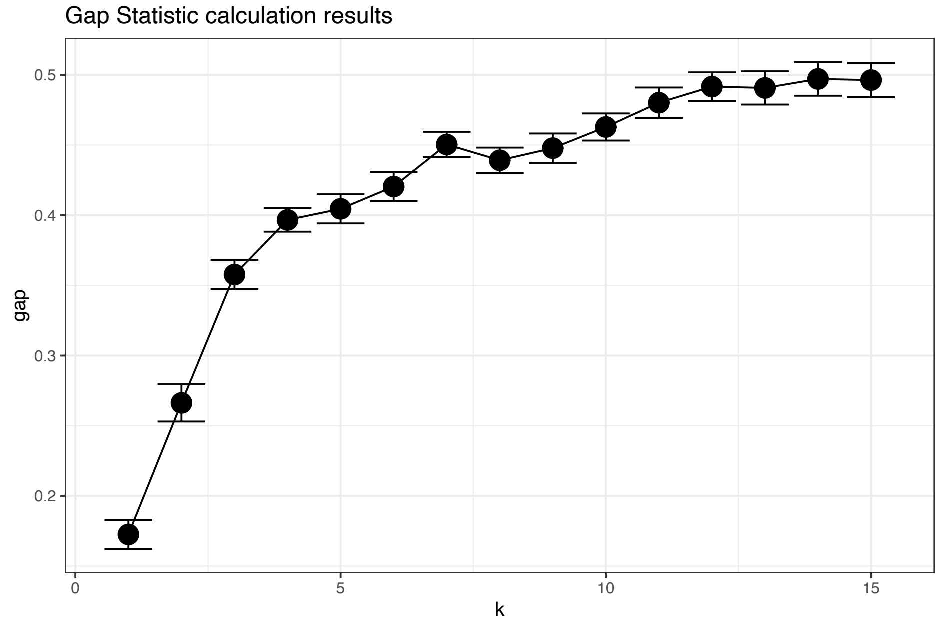

Supplementary Fig. S7: Gap statistic calculation results with number of clusters (k) against goodness of clustering measure (gap).

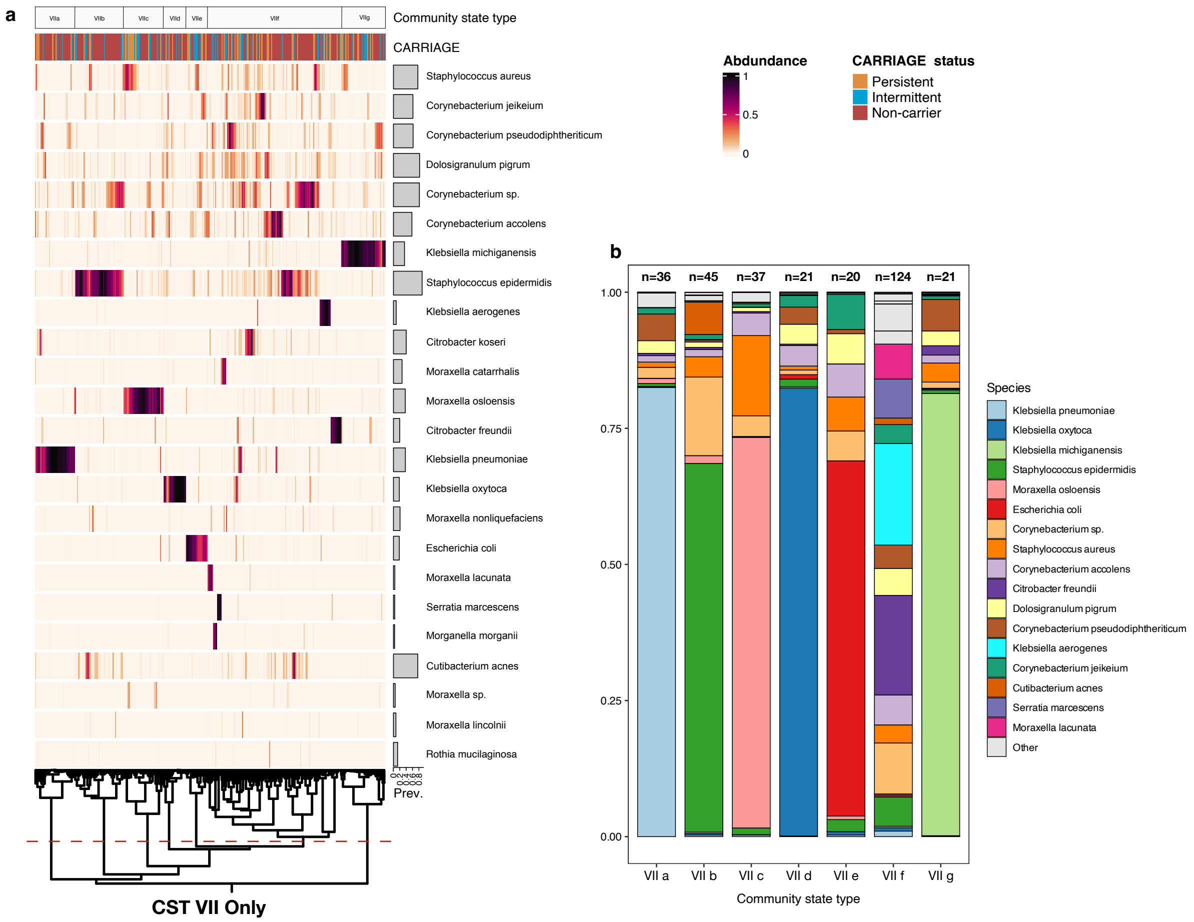

Supplementary Fig. S8: Sub-clusters and associated mean species abundance observed in CST-VII (a) sub-set plot of CST-VII alone from Figure 2A. Samples are ordered by hierarchical clustering using Bray-Curtis distances based on the compositional, relative abundance data, represented by the dendrogram. Prevalence of each species across the samples is represented by the horizontal bar plots. Community state types (CSTs) and *S. aureus* colonisation status of samples are represented above the heatmap. Sub-clusters are visually defined by partitioning the samples based on the dendogram (dashed red-line) **(b)** Mean microbial composition of samples for each CST-VII sub-cluster at a species level. Sub-clusters are dominated by a single species, revealing additional structure within the nasal microbiome across the sampled population. For (a, b), n=324 samples from unique individuals.

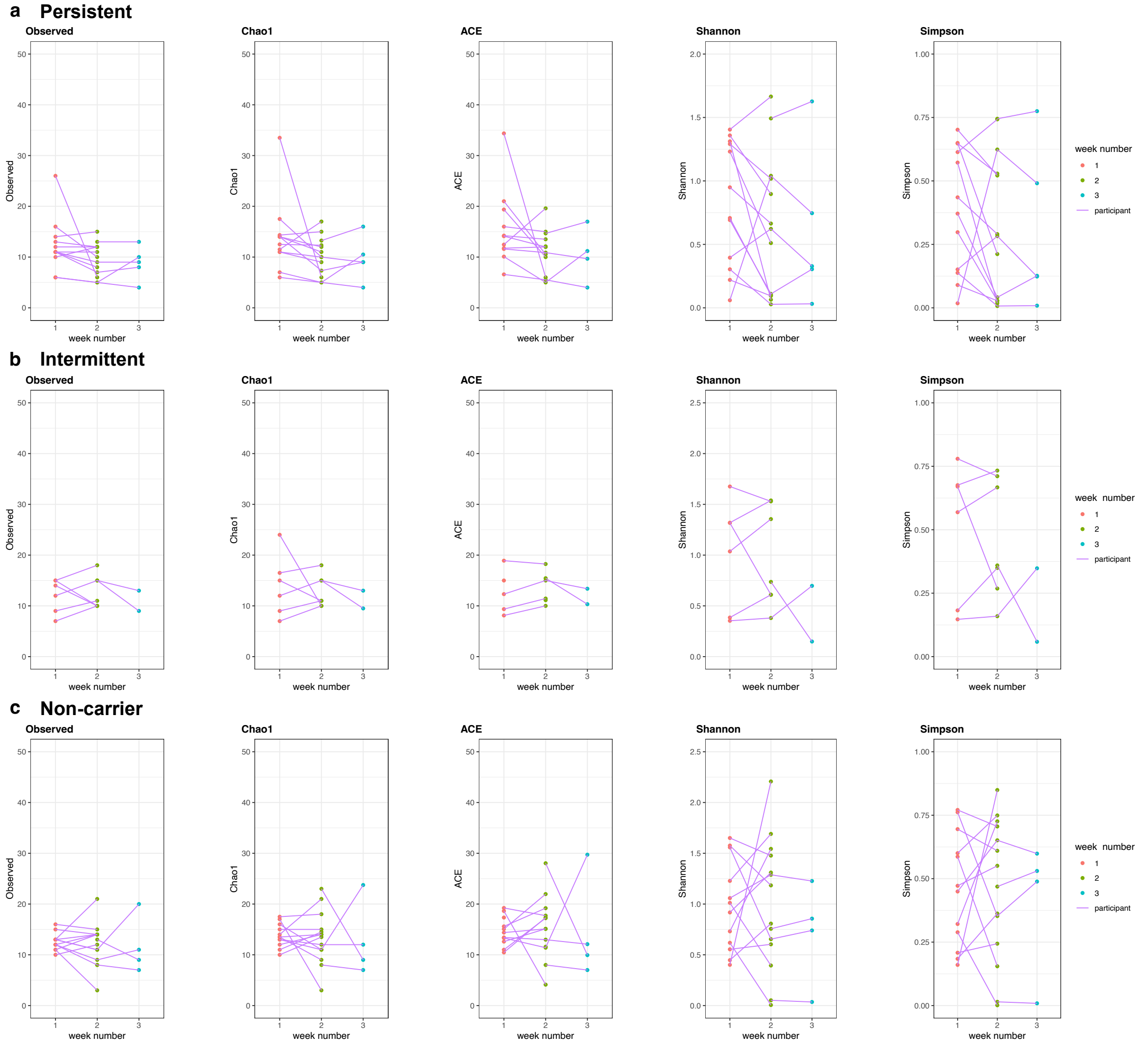

Supplementary Fig. S9: Alpha diversity of serially sampled study participants by colonisation status. Alpha diversity is presented by multiple indices by carriage state (**a**) Persistent (**b**) Intermittent (**c**) non-carriers. Plot comparing alpha diversity of anterior nares by samples week number (one, two, or three) where available. Alpha diversity is calculated after rarefication of high-quality reads. N=34 individual participants, where two or three samples (n=75) were available over consecutive weeks; persistent carriers (n=13), intermittent carriers (n=7), and non-carriers (n=14).

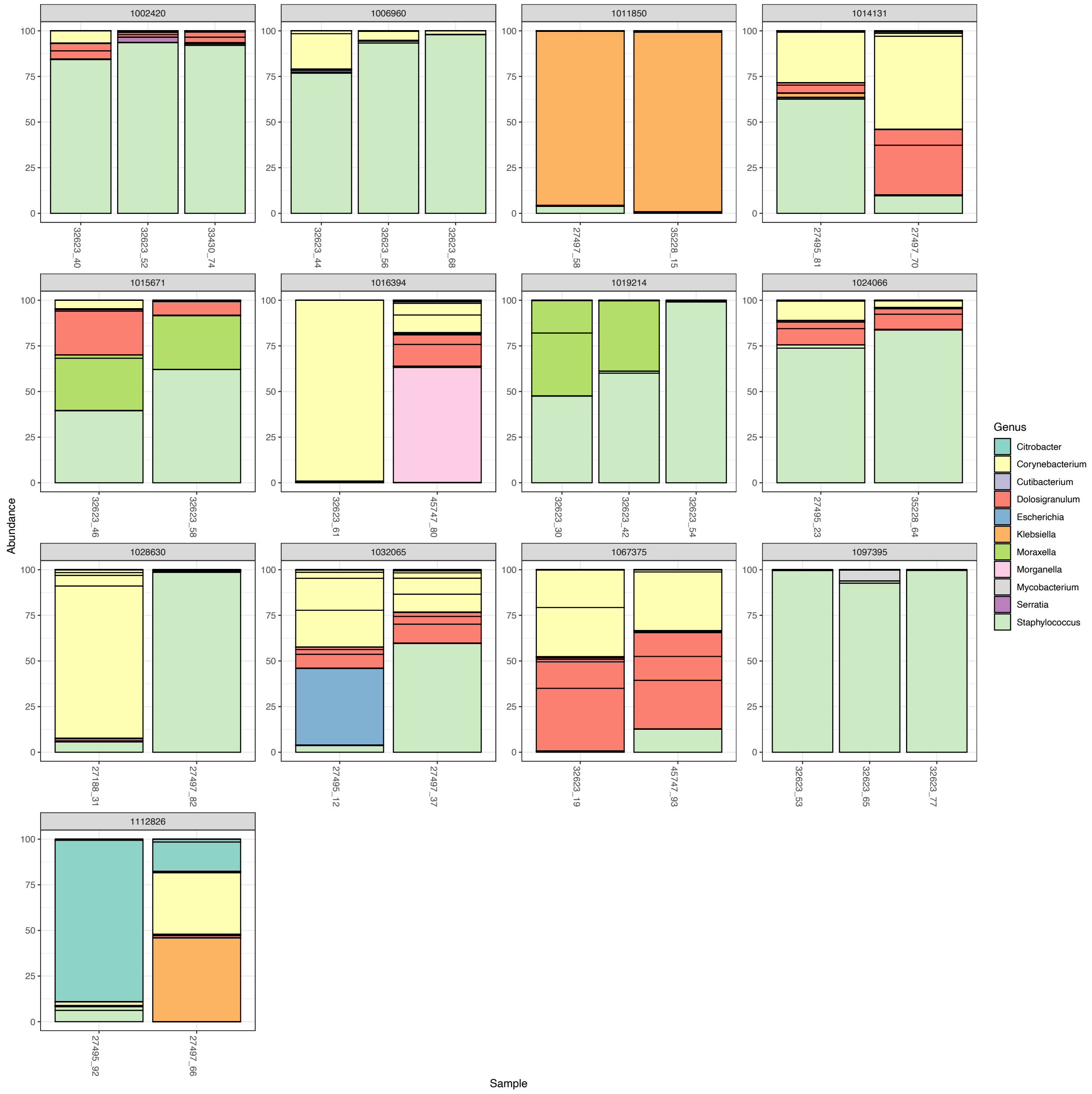

Supplementary Fig. S10: Relative abundance of serially sampled *Staphylococcus aureus* persistent carriers at a genus level. Relative abundance is calculated after rarefication of high-quality reads. N=13 individual participants.

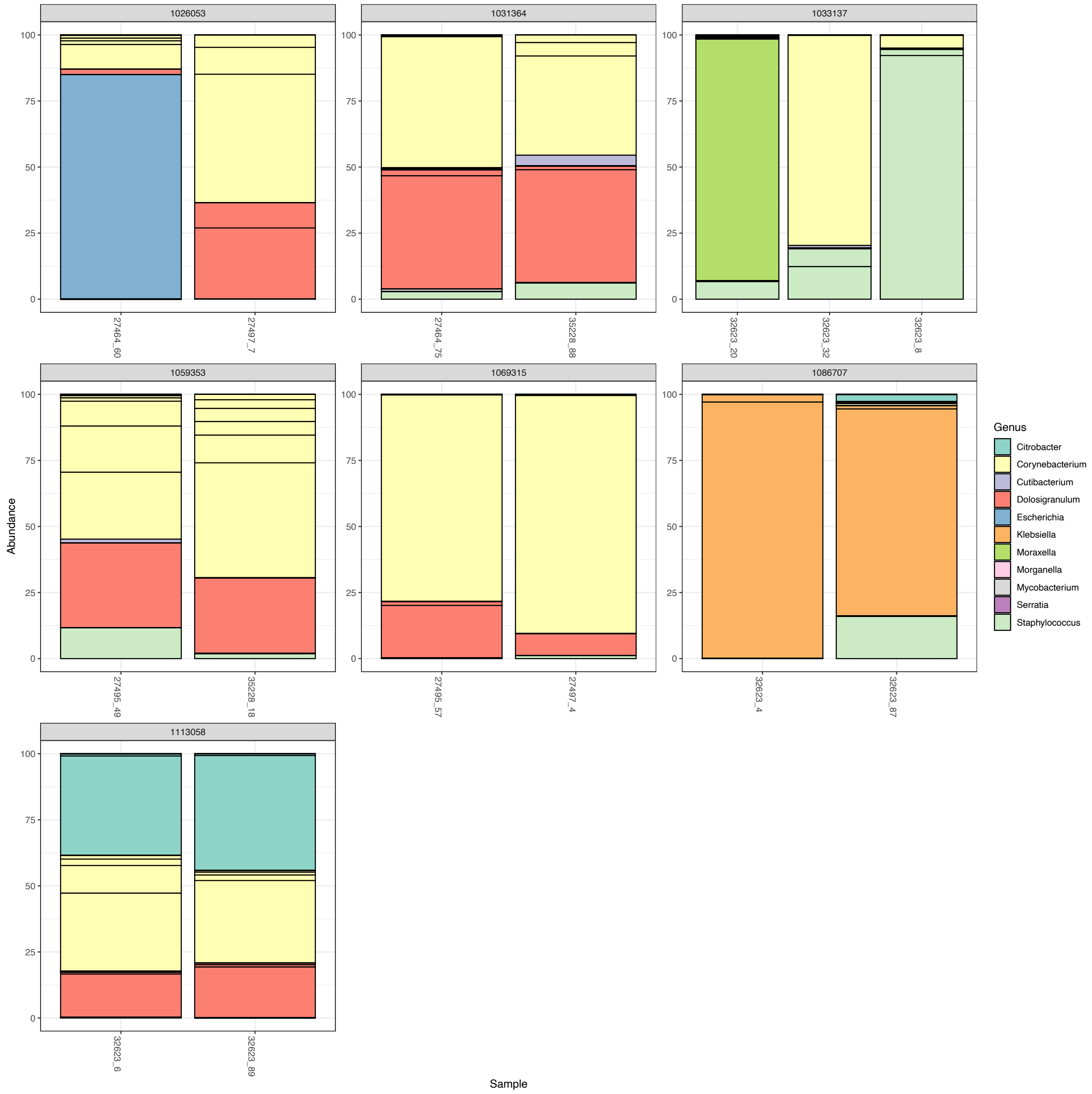

Supplementary Fig. S11: Relative abundance of serially sampled *Staphylococcus aureus* intermittent carriers at a genus level. Relative abundance is calculated after rarefication of high-quality reads. N=7 individual participants.

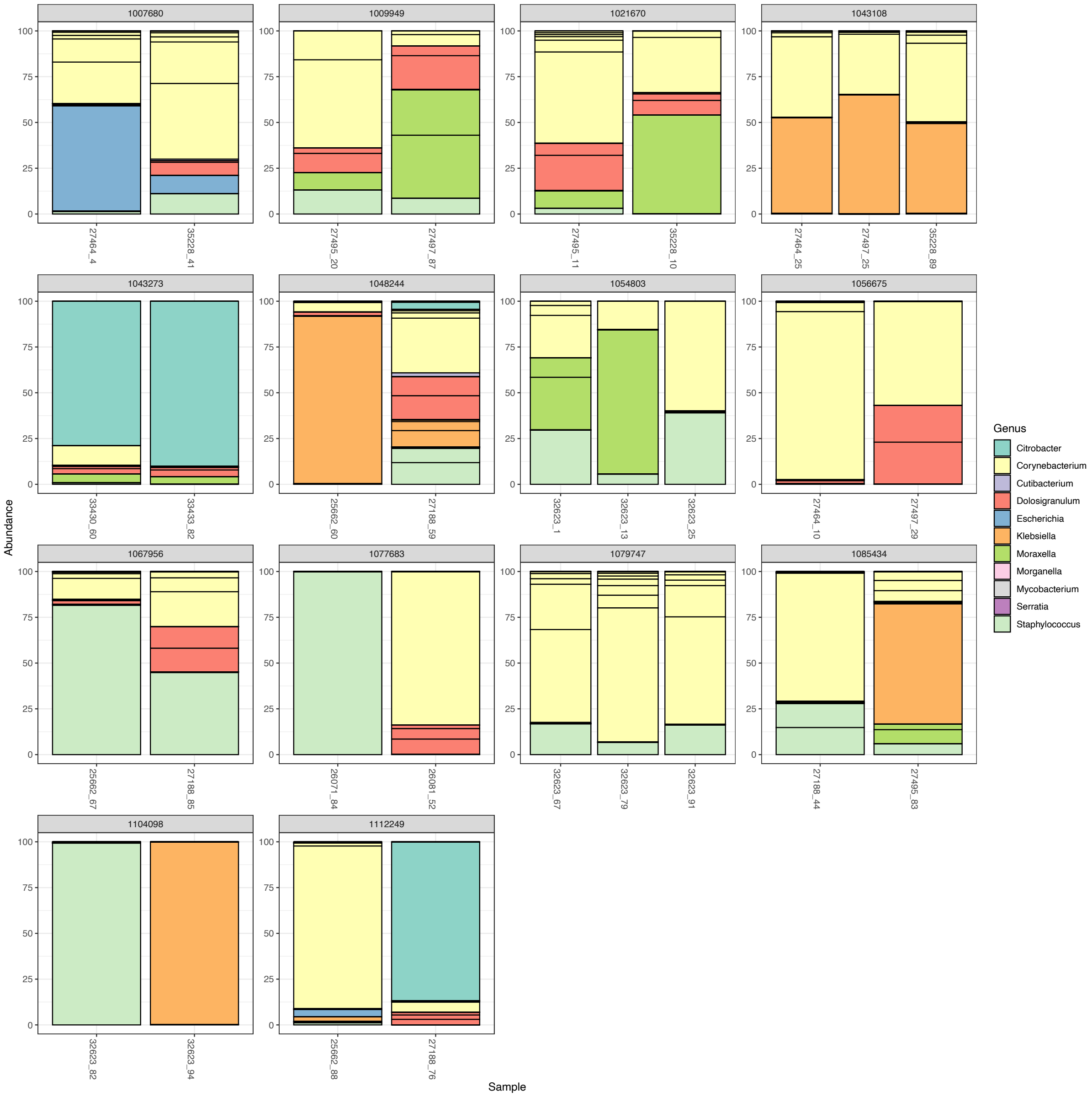

Supplementary Fig. S12: Relative abundance of serially sampled *Staphylococcus aureus* non-carriers at a genus level. Relative abundance is calculated after rarefication of high-quality reads. N=14 individual participants.

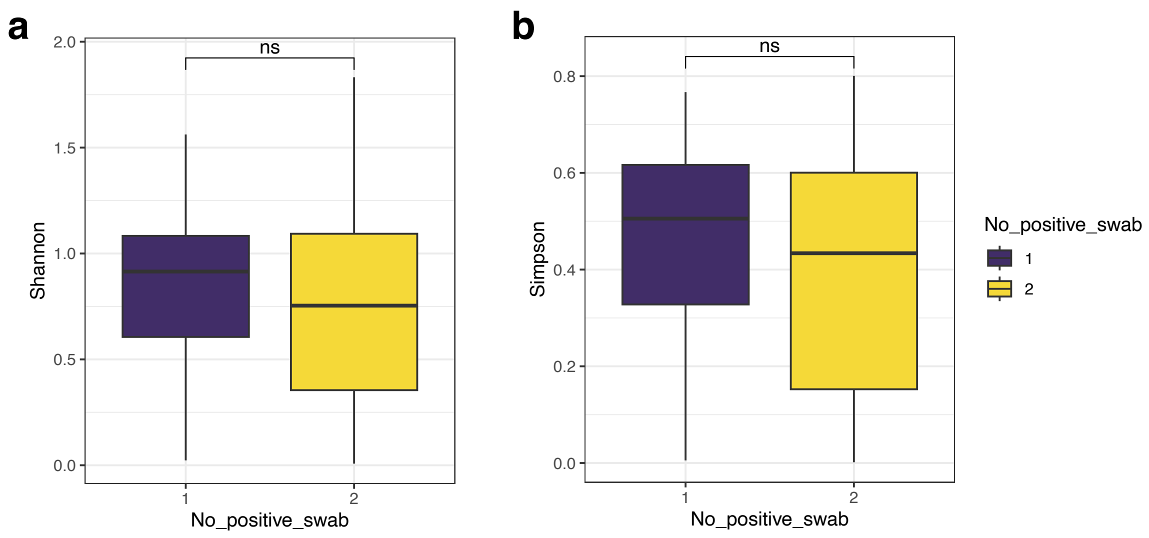

Supplementary Fig. S13: Alpha diversity from nasal samples by frequency of nasal swabs positive for *Staphylococcus aureus* amongst intermittent carriers. **(a-b)** Box plot comparing alpha diversity (Shannon and Simpson) from nasal samples by the number of swabs positive (one or two) for *Staphylococcus aureus* from intermittent carriers; n=169 samples from unique individuals. **(a)** Shows no significant difference in alpha diversity (Shannon) between intermittent carriers with one swab positive for *S. aureus* vs those with two swab (p=0.21). **(b)** Shows no significant difference in alpha diversity (Simpson) between intermittent carriers with one swab positive for *S. aureus* vs those with two swab (p=0.19). 'ns', 'non-significant'.

Supplementary Results

Random Forest model performance of combined dataset

We assessed the performance of the random forest model to predict carriage status with the combined dataset including all samples above 500 reads and those rarefied above 10,000.

We determined the best number of candidates to be sampled at each tree (mtry) to be 4. The estimated test classification accuracy of the trained model was 70.6% (1-estimated out of box error). We determined the accuracy of the model with the test data. The overall accuracy of the model was 69.9% (C.I.= 62.3-76.2%, p=0.001) significantly exceeding the no information rate.
