# Supplementary material for: Large-scale characterisation of the nasal microbiome redefines Staphylococcus aureus colonisation status
Source: Nat Commun. 2025 Dec 2;16:10415. doi: 10.1038/s41467-025-66564-4 (PMC12672568; doi:10.1038/s41467-025-66564-4)
Supplement: Supplementary file 2 — Description of Additional Supplementary Files [file 41467_2025_66564_MOESM2_ESM.pdf]

Description of Additional Supplementary Data for Aggarwal et al

File Name: Supplementary Data 1
Description: Sample accessions of 16S rRNA sequences used in this study.

File Name: Supplementary Data 2
Description: Metadata and ENA accessions for *S. aureus* isolates.

File Name: Supplementary Data 3
Description: Operational Taxonomic Unit (OTU) table of unadjusted read counts generated from 16S rRNA gene sequencing of samples. We clustered high-quality unique sequences with Oligotyping v2.1 (-M option to 1000), which were assigned to NODES, and referred to as OTUs, with the 'Minimum Entropy Decomposition' (MED) option. The column headers have corresponding taxonomic classification in Supplementary Table S10.

File Name: Supplementary Data 4
Description: Taxonomic classification of each Operational Taxonomic Unit (OTU). We created a customised silva SSU Ref database (NR99, release 132), where we removed the majority of environmental and uncultured taxa, and carried out taxonomic assignment with ARB (v6.0.6-3). In some instances, where a mismatch was observed within the taxonomic groups, we assigned taxa to the OTU sequence with BLAST (Supplementary Table S2).
