# Supplementary material for: Large-scale characterisation of the nasal microbiome redefines Staphylococcus aureus colonisation status
Source: Nat Commun. 2025 Dec 2;16:10415. doi: 10.1038/s41467-025-66564-4 (PMC12672568; doi:10.1038/s41467-025-66564-4)
Supplement: Supplementary file 7 — Reporting Summary [file 41467_2025_66564_MOESM7_ESM.pdf]

nature portfolio

Corresponding author(s): Dinesh Aggarwal
Ewan Harrison

Last updated by author(s): 06/11/2025

Reporting Summary

Nature Portfolio wishes to improve the reproducibility of the work that we publish. This form provides structure for consistency and transparency in reporting. For further information on Nature Portfolio policies, see our Editorial Policies and the Editorial Policy Checklist.

Please do not complete any field with "not applicable" or n/a. Refer to the help text for what text to use if an item is not relevant to your study.
For final submission: please carefully check your responses for accuracy; you will not be able to make changes later.

Statistics

For all statistical analyses, confirm that the following items are present in the figure legend, table legend, main text, or Methods section.

n/a	Confirmed	
[ ]	[x]	The exact sample size (*n*) for each experimental group/condition, given as a discrete number and unit of measurement
[x]	[ ]	A statement on whether measurements were taken from distinct samples or whether the same sample was measured repeatedly
[ ]	[x]	The statistical test(s) used AND whether they are one- or two-sided<br>*Only common tests should be described solely by name; describe more complex techniques in the Methods section.*
[ ]	[x]	A description of all covariates tested
[ ]	[x]	A description of any assumptions or corrections, such as tests of normality and adjustment for multiple comparisons
[ ]	[x]	A full description of the statistical parameters including central tendency (e.g. means) or other basic estimates (e.g. regression coefficient) AND variation (e.g. standard deviation) or associated estimates of uncertainty (e.g. confidence intervals)
[ ]	[x]	For null hypothesis testing, the test statistic (e.g. *F*, *t*, *r*) with confidence intervals, effect sizes, degrees of freedom and *P* value noted<br>*Give P values as exact values whenever suitable.*
[x]	[ ]	For Bayesian analysis, information on the choice of priors and Markov chain Monte Carlo settings
[x]	[ ]	For hierarchical and complex designs, identification of the appropriate level for tests and full reporting of outcomes
[ ]	[x]	Estimates of effect sizes (e.g. Cohen's *d*, Pearson's *r*), indicating how they were calculated

Our web collection on statistics for biologists contains articles on many of the points above.

Software and code

Policy information about availability of computer code

Data collection	No software was used for data collection
Data analysis	For 16S rRNA gene sequencing, per experiment, an equimolar pool of PCR libraries was sequenced at the Wellcome Sanger Institute in-house sequencing facility, using the Illumina MiSeq (300bp paired-end reads, v3 Reagent Kit). For whole-genome sequencing, S. aureus isolates were sequenced at the Wellcome Sanger Institute with 96 sample libraries sequenced on a 300bp PE MiSeq lane (with a 1% PhiX spike).<br><br>We used a modified mothur MiSeq standard operating procedure (SOP) to process paired fastq files (MOTHUR wiki at http://www.mothur.org/wiki/MiSeq_SOP). The Silva bacterial database 'silva.nr_v132.align' was used to align quality-screened sequences and chimeras removed using Uchime. We clustered high-quality unique sequences with Oligotyping v2.1 and carried out taxonomic assignment with ARB (v6.0.6-3). In some instances, where a mismatch was observed within the taxonomic groups, we assigned taxa to the OTU sequence with BLAST (see Supplementary Table S2). We then combined the output in R (v4.4.1) into a phyloseq object for onward analysis. We used Decontam v1.16.0 to account for laboratory negative controls.<br>We conducted microbial diversity and compositional analysis in R using diversity indices calculated with the phyloseq (v1.40) and vegan (v2.6-4) packages. We manipulated data in Excel 2016 and R (v4.4.1). We manipulated data in Excel 2016 and R (v4.4.1). We generated figures using ggplot2 (v3.4.0), phyloseq (v1.40), ComplexHeatmap (v2.24.1), microViz (v0.11.0), and ggtree (v3.16.3). To determine the number of clusters in the data, we calculated a gap statistic with ordination values using Bray-Curtis distances, using the R package 'cluster' function clusGap(). We used ANCOM-BC (v1.6.4) to evaluate differential abundance of microbial species in the study groups. Species-level networks were inferred with NetCoMi (v1.2). The network was visualised with igraph (v2.1.4). We used the R package randomForest (v4.7-1.1) to fit a random forest classifier for carriage status. We obtained sensitivity and specificity values<br>of the model with the R package caret (v6.0-90) whilst receiver operating characteristic curve (ROC) curves and AUC were obtained with the R package pROC (v1.18.4).<br>[illegible]

(github.com/avantonder/bacQC). Species classification for each sample was performed using Kraken and Bracken. Using the nextflow pipeline, assembleBAC (github.com/avantonder/assembleBAC), we produced annotated assemblies with trimmed fastqs. The pipeline uses shovill (v1.1.0) for assembly. We annotated assemblies with prokka (v.1.14.5) using a genus-specific database from RefSeq for annotation. QC metrics were summarised by MultiQC and the additional QC metrics generated by Panaroo(v1.3.4). We assigned sequence types (STs) with mlst (v2.19.0) (github.com/tseemann/mlst), and where these were not assigned, assemblies we queried the sequences on the PubMLST web server. We produced core-genome alignments with Panaroo (v 1.3.4) with a core-genome threshold set to 98%. We extracted variant sites from the core-genome alignment with snp-sites (v2.5.1). We used IQ-TREE (v2.1.2) to estimate phylogenetic trees with optimal trees determined by ModelFinder.

For manuscripts utilizing custom algorithms or software that are central to the research but not yet described in published literature, software must be made available to editors and reviewers. We strongly encourage code deposition in a community repository (e.g. GitHub). See the Nature Portfolio guidelines for submitting code & software for further information.

Data

Policy information about availability of data

All manuscripts must include a data availability statement. This statement should provide the following information, where applicable:

- Accession codes, unique identifiers, or web links for publicly available datasets
- A description of any restrictions on data availability
- For clinical datasets or third party data, please ensure that the statement adheres to our policy

All sequencing data is publicly available in the European Nucleotide Archive, with details outlined in Supplementary Table S6 and S7. Unique sequences ('Nodes') generated from the bioinformatics pipeline used for taxonomic assignment can be access online (DOI: https://doi.org/10.5281/zenodo.17160106).

Research involving human participants, their data, or biological material

Policy information about studies with human participants or human data. See also policy information about sex, gender (identity/presentation), and sexual orientation and race, ethnicity and racism.

Reporting on sex and gender

Self-reported biological sex was available through the study cohort metadata, and included in multiple analyses (variation in diversity, S aureus abundance, and community state type association. Additional participant relevant metadata was obtained through questionnaires – metadata available for use in analyses included smoking, pet ownership, healthcare contact, chronic skin condition, asthma, allergies, and diabetes.

Disaggregated data on sex and gender is not available.

Reporting on race, ethnicity, or other socially relevant groupings

Please specify the socially constructed or socially relevant categorization variable(s) used in your manuscript and explain why they were used. Please note that such variables should not be used as proxies for other socially constructed/relevant variables (for example, race or ethnicity should not be used as a proxy for socioeconomic status).
Provide clear definitions of the relevant terms used, how they were provided (by the participants/respondents, the researchers, or third parties), and the method(s) used to classify people into the different categories (e.g. self-report, census or administrative data, social media data, etc.)
Please provide details about how you controlled for confounding variables in your analyses.

Population characteristics

To study the biological basis of S. aureus colonisation we sampled generally healthy adult blood donors (see recruitment) from across England with three self-taken nasal swabs taken at weekly intervals. S. aureus colonisation status was assessed by culture, and was defined as: (i) persistent colonisation (306/1091 (28.0%); three S. aureus positive weekly nasal swabs, (ii) intermittent colonisation 191/1091 (17.5%); one or two positive swabs, and (iii) non-carrier 594/1091 (54.4%): no positive swabs (89 failed to return all samples). Participants had a mean age of 51.4 (median, 53) and 52.8% were female. A total of 1,756 samples, which included the first swabs of 1,180 participants underwent 16S rRNA gene sequencing to determine the microbiome composition.

Recruitment

Participants of the CARRIAGE study have been recruited from three nationwide blood donor cohorts in England, United Kingdom (INTERVAL, COMPARE and STRIDES). When donors originally consented to INTERVAL, COMPARE and STRIDES they agreed to being contacted in the future about joining other studies. The first wave of recruitment into CARRIAGE involved participants from the INTERVAL study, a multi-purpose epidemiological bioresource which commenced as a randomised trial of blood donation frequency (http://www.intervalstudy.org.uk). The second wave involved participants from the COMPARE study (http://www.comparestudy.org.uk) which aimed to compare different methods for the measurement of haemoglobin levels in blood donors. The third wave involved participants from the STRIDES (STRategies to Improve Donor ExperienceS) study (http://www.strides-study.org.uk), the bioresource component of a cluster-randomised trial in England assessing different interventions to prevent vasovagal reactions (fainting or feeling faint) during routine blood donation. Participants in the three source blood donor cohorts were recruited from blood donation sites (mobile teams and donor centres) of National Health Service Blood and Transplant (NHSBT) across England. Participants were eligible to join the study if they: had been part of the INTERVAL, COMPARE or STRIDES studies; had a working email address and internet connection; resided in mainland England; and had a good understanding of the English language, both written and oral (study materials were not tailored to support non-English language speakers). Exclusion criteria from the study included: having received three or more invites to join other studies in the last year; having withdrawn consent from the INTERVAL, COMPARE or STRIDES studies, any abnormalities or on-going medical conditions that affect the nose (e.g. nosebleeds, nasal polyps, rhinitis, etc.); having undergone nasal surgery in the last year; having had a broken nose in the last year; or if the participants would be unavailable to participant in the study within two months of the invitation date. This cohort represents healthy human adults from the community, and the data is therefore not representative of the child nasal microbiome or that of people ineligible for blood donation.

Ethics oversight	The CARRIAGE study protocol was approved by the National Research Ethics Service Committee North-West - Lancaster Research Ethics Committee, 27/06/2016, REC reference: 16/NW/0507, IRAS project ID: 202688. All participants provided informed consent. The study is registered at ISRCTN: ISRCTN10474633.

Note that full information on the approval of the study protocol must also be provided in the manuscript.

Field-specific reporting

Please select the one below that is the best fit for your research. If you are not sure, read the appropriate sections before making your selection.

☒ Life sciences ☐ Behavioural & social sciences ☐ Ecological, evolutionary & environmental sciences

For a reference copy of the document with all sections, see nature.com/documents/nr-reporting-summary-flat.pdf

Life sciences study design

All studies must disclose on these points even when the disclosure is negative.

Sample size	The study is a descriptive epidemiological study and observational in nature. It represents the largest analysis of nasal microbiome data, where individuals have been serially cultured from their anterior nares to determine their S. aureus colonisation status. There were no sample size calculations conducted.
Data exclusions	The vast majority of available data was included in the study. Software Mothur was used to quality control 16S rRNA sequences. The Silva bacterial database 'silva.nr_v132.align' was used to align quality-screened sequences and chimeras removed using Uchime. Sequences were then classified using the same Silva reference database and the Silva taxonomy database 'silva.nr_v132.tax', with the removal of chloroplast, mitochondria, unknown, and eukaryota sequences. During taxonomic assignment we removed the majority of environmental and uncultured taxa with ARB (v6.0.6-3).<br>We systematically identified and removed contaminants, and accounted for variability in sequencing depth. We identified contaminants and removed these by identifying batch effects and accounting for negative controls. Batch effects were assessed by calculating the spearman's correlation co-efficient of species against each location of extraction, and location of PCR reaction. We then examined correlation of species with sample DNA concentrations. We used well characterised 'kitome' and environment contaminants to identify additional associated contaminants by calculating 'species-species' correlation coefficients. We used Decontam v1.16.0 to account for laboratory negative controls, run with the 'isnotcontam' function and with each sequencing run provided as a batch (Supplementary Fig. S3 and S4 and Supplementary Table S3). We determined a suitable rarefication depth of 10,000 reads using rarefication curves and examining the read depth at which the majority of sample taxa numbers plateaued (Supplementary Fig. S5). We removed species with an abundance of less than 0.1% across samples, below which we expected the removal of most contaminants and account for the variability in rare species composition between runs. For diversity analyses, the rarified dataset was used. For abundance analyses, to mitigate data loss, we combined samples with greater than 500 high quality reads with samples that had greater than 10000 reads and rarefied. Unless stated otherwise, de-duplicated data was used, and the sample with the greatest number of reads used from each individual.<br><br>For whole-genome sequencing, we discarded samples with less than 90% reads matching to S. aureus and those with <30x coverage from onward analyses. Assemblies with an N50 value <10000, length of less than 2.6Mbp or greater than 3.0Mbp, or with a spuriously high number of contigs summarised by MultiQC and the QC metrics generated by Panaroo(v1.3.4) were removed from onward analyses. Samples with greater than 300 contigs were found to be outliers.
Replication	To examine consistency of microbiome data across weeks from single individuals, we sequence multiple replicates from individuals - which provides confidence that the taxa observed are consistently sequenced and observed. To examine the robustness of microbial associations with S aureus colonisation states, we have conducted multiple sensitivity analyses which corroborate findings. We have run version controlled software code on multiple occasions to replicate all results, whilst two separate researchers have independently examined 16S rRNA sequence data to confirm taxonomic identification and diversity anaylses. All sequencing data is publicly available to enable other researchers to replicate findings.
Randomization	This study reports observational microbiome data derived from prospectively sampled cohorts (see recruitment). The study describes the microbial constituents of the anterior nares in relation to the longitudinal carriage of S aureus, and therefore randomisation is not appropriate.
Blinding	The study is an observational cohort and carriage groups were defined from culture results collected during follow-up, so group allocation could not be blinded at recruitment. Laboratory processing and early-stage bioinformatics were performed with indexed raw sequence data, so for taxa assignment and contamination removal, staff were unaware of carriage status. Downstream statistical analyses to identify the ecological basis for carriage followed a pre-specified plan and required carriage status to be integrated with microbiome data.

Reporting for specific materials, systems and methods

We require information from authors about some types of materials, experimental systems and methods used in many studies. Here, indicate whether each material, system or method listed is relevant to your study. If you are not sure if a list item applies to your research, read the appropriate section before selecting a response.

Materials & experimental systems

n/a	Involved in the study
☒	☐ Antibodies
☒	☐ Eukaryotic cell lines
☒	☐ Palaeontology and archaeology
☒	☐ Animals and other organisms
☒	☐ Clinical data
☒	☐ Dual use research of concern
☒	☐ Plants

Methods

n/a	Involved in the study
☒	☐ ChIP-seq
☒	☐ Flow cytometry
☒	☐ MRI-based neuroimaging

Plants

Seed stocks	N/A
Novel plant genotypes	N/A
Authentication	N/A

This checklist template is licensed under a Creative Commons Attribution 4.0 International License, which permits use, sharing, adaptation, distribution and reproduction in any medium or format, as long as you give appropriate credit to the original author(s) and the source, provide a link to the Creative Commons license, and indicate if changes were made. The images or other third party material in this article are included in the article's Creative Commons license, unless indicated otherwise in a credit line to the material. If material is not included in the article's Creative Commons license and your intended use is not permitted by statutory regulation or exceeds the permitted use, you will need to obtain permission directly from the copyright holder. To view a copy of this license, visit http://creativecommons.org/licenses/by/4.0/
